# Synoviolin meets metabolic disorders

**DOI:** 10.1186/ar3578

**Published:** 2012-02-09

**Authors:** Naoko Yagishita, Satoko Aratani, Daisuke Hasegawa, Yoshihisa Yamano, Kusuki Nishioka, Toshihiro Nakajima

**Affiliations:** 1 Institute of Medical Science, St Marianna University School of Medicine, Kawasaki, Kanagawa 216-8512, Japan; 2 Institute of Medical Science, Tokyo Medical University, Shinjuku-ku, Tokyo 160-8402, Japan; 3 Bayside Misato Medical Center, Kochi 781-0112, Japan

## 

Rheumatoid arthritis (RA) is consists of multiple processes such as chronic inflammation, overgrowth of synovial cells, joint destruction and fibrosis. To clarify the mechanism of outgrowth of synovial cells, we carried out immunoscreening using anti-rheumatoid synovial cell antibody, and cloned 'Synoviolin' [1]. Synoviolin is endoplasmic reticulum (ER)-resident E3 ubiquitin ligases, and is involved in ER-associated degradation (ERAD). Synoviolin is highly expressed in synoviocytes of patients with RA. Overexpression of synoviolin in transgenic mice leads to advanced arthropathy caused by reduced apoptosis of synoviocytes [1]. We postulate that the hyperactivation of the ERAD pathway by overexpression of synoviolin results in prevention of ER-stress-induced apoptosis leading to synovial hyperplasia [2]. In addition, Synoviolin ubiquitinates and sequesters the tumor suppressor p53 in the cytoplasm, thereby negatively regulating its biological functions [3]. Therefore Synoviolin regulates, not only apoptosis in response to ER stress, but also a p53-dependent apoptotic pathway. These studies indicate that Synoviolin is involved in overgrowth of synovial cells through its anti-apoptotic effects. Further analysis showed that Synoviolin is also involved in fibrosis among the multiple processes [4]. Therefore, it was suggested that Synoviolin is thought to be a candidate for pathogenic factor for arthropathy through its involvement of multiple processes.

As for the treatment of RA, biological agents are approved for clinical use, and these drugs have dramatically changed the treatment of RA during the past decade. However, in some cases patients fail to respond to the biologic treatment or adverse effects develop such as; an increased risk of infections. It was reported that elevated Synoviolin levels were identified in circulating monocytes and were associated with nonresponse to infliximab treatment. Moreover, these agents are associated with high costs and discomfort arising from subcutaneous or intravenous administration. Thus, there is a clear need for the development of cheaper, orally administrated therapies with fewer side effects. Then, we successfully discovered Synoviolin inhibitors. We are now proceeding with the optimization of small compounds, and we hope our research will lead to the development of a new therapy for RA and serve as an example of the therapeutic benefit of developing E3 ligase inhibitors.

In addition, to clarify the physiological function of Synoviolin in adult, we recently generate synoviolin conditional knockout mice using tamoxifen inducible Cre transgenic mice under CAG promoter. In today's session, I'd like to introduce the preliminary data of synoviolin conditional knockout mice.
